# The effects of probiotic administration on patients with prediabetes: a meta-analysis and systematic review

**DOI:** 10.1186/s12967-022-03695-y

**Published:** 2022-11-02

**Authors:** Ya Li, You Wu, Lili Wu, Lingling Qin, Tonghua Liu

**Affiliations:** Key Laboratory of Health Cultivation of the Ministry of Education, Beijing University of Chinese Medicine, 100029 Beijing, China

**Keywords:** Prediabetes, Probiotics, Random control trials, Meta-analysis, Systematic review

## Abstract

**Background:**

This paper aimed to examine the effects of probiotics on eight factors in the prediabetic population by meta-analysis, namely, fasting blood glucose (FBG), glycated haemoglobin A1c (HbA1c), homeostatic model assessment of insulin resistance (HOMA-IR), quantitative insulin sensitivity check index (QUICKI), total cholesterol (TC), triglyceride (TG), high-density lipoprotein cholesterol (HDL-C) and low-density lipoprotein cholesterol (LDL-C), and the mechanisms of action are summarized from the existing studies.

**Methods:**

Seven databases (PubMed, Web of Science, Embase, Cochrane Library, SinoMed, CNKI, and Wanfang Med) were searched until March 2022. Review Manager 5.4 was used for meta-analysis. The data were analysed using weighted mean differences (WMDs) or standardized mean differences (SMDs) under a fixed effect model to observe the efficacy of probiotic supplementation on the included indicators.

**Results:**

Seven publications with a total of 460 patients were included. According to the meta-analysis, probiotics were able to significantly decrease the levels of HbA1c (WMD, -0.07; 95% CI -0.11, -0.03; P = 0.001), QUICKI (WMD, 0.01; 95% CI 0.00, 0.02; P = 0.04), TC (SMD, -0.28; 95% CI -0.53, -0.22; P = 0.03), TG (SMD, -0.26; 95% CI -0.52, -0.01; P = 0.04), and LDL-C (WMD, -8.94; 95% CI -14.91, -2.97; P = 0.003) compared to levels in the placebo group. The effects on FBG (WMD, -0.53; 95% CI -2.31, 1.25; P = 0.56), HOMA-IR (WMD, -0.21; 95% CI -0.45, 0.04; P = 0.10), and HDL-C (WMD, 2.05; 95% CI -0.28, 4.38; P = 0.08) were not different from those of the placebo group.

**Conclusion:**

The present study clearly indicated that probiotics may fulfil an important role in the regulation of HbA1c, QUICKI, TC, TG and LDL-C in patients with prediabetes. In addition, based on existing studies, we concluded that probiotics may regulate blood glucose homeostasis in a variety of ways.

**Trial Registration:**

This meta-analysis has been registered at PROSPERO with ID: CRD42022321995.

## Background

Diabetes and its complications are among the chronic noncommunicable diseases that pose a serious threat to public health [1]. Prediabetes is a period of impaired glucose regulation that includes impaired fasting glucose and impaired glucose tolerance with elevated blood glucose levels [2] that do not yet meet the diagnostic criteria for diabetes [3]. The prevalence of prediabetes is increasing each year [4] and is much higher than that of type 2 diabetes [5]. According to statistics, 70% of patients with prediabetes eventually develop diabetes [3]. In the treatment of prediabetes, lifestyle improvement and drug therapy have limitations and side effects, respectively [3]. In this light, there is an urgent need for natural and safe strategies to control and delay the progression of prediabetes to diabetes [6].

However, prediabetes remains a reversible stage in clinical practice [7–9]. Recent studies have found certain mechanisms mediating the development from the prediabetic stage to diabetes. One of the important changes that occur in the process is the alteration of the gut microbiota, which affects intestinal permeability, metabolic regulation and insulin resistance [10].

Probiotics exert beneficial effects on the body by regulating the intestinal microbiota [11]. An elevated abundance of intestinal flora is associated with remission of diabetes. For example, in some studies, probiotics have been shown to improve insulin resistance, regulate blood glucose homeostasis, lower blood lipids, and delay or inhibit the onset of diabetes and its complications [12–16]. However, the mechanisms of the role of probiotics in prediabetes are not fully understood. Moreover, there are also inconsistent views on the beneficial effects of probiotics. Some studies have found that *Lactobacillus casei* and *Lactobacillus rhamnosus* HN001 have a limited effect on glucolipid metabolism in prediabetes [17, 18]. Accordingly, we performed the present meta-analysis to determine whether probiotics are beneficial in prediabetes and to discuss their mechanisms of action on the basis of existing studies.

The PICO principle was adopted in this paper, namely, participants, intervention, comparison, and outcome. The specific factors are as follows: P – people with prediabetes; I – probiotics given orally only and unlimited types and forms; C – equal doses of placebo; and O – primary indicators of fasting blood glucose (FBG) and glycated haemoglobin (HbA1c), and secondary indicators of homeostatic model assessment of insulin resistance (HOMA-IR), quantitative insulin sensitivity check index (QUICKI), total cholesterol (TC), triglyceride (TG), high-density lipoprotein cholesterol (HDL-C) and low-density lipoprotein cholesterol (LDL-C).

## Materials and methods

### Search strategy

This meta-analysis and systematic review were performed according to the Preferred Reporting Items for Systematic Reviews and Meta-Analyses (PRISMA) statement [19].

Seven databases (PubMed, Web of Science, Embase, Cochrane Library, SinoMed, CNKI [China National Knowledge Infrastructure], and Wanfang Med) were searched from inception to March 2022. The search terms were as follows: [(probiotic agent) OR (gastrointestinal microbiota) OR (gut dysbiosis) OR (gut microbiota) OR (gut microbiome) OR (probiotics)] AND [(prediabetes) OR (prediabetic states) OR (states, prediabetic) OR (state, prediabetic) OR (impaired glucose regulation) OR (impaired glucose tolerance) OR (impaired fasting glucose) OR (impaired glucose metabolism) OR (abnormal glucose metabolism) OR (prediabetic state)] AND [(randomized controlled trial OR randomized OR placebo)]. Finally, corresponding to the database mentioned above, we retrieved n = 42, 58, 49, 174, 25, 12, 49 papers respectively, for a total of 409 articles.

### Study selection

#### Inclusion criteria

Only randomized controlled trials of probiotics for prediabetes were selected. Among them, the probiotics group only used probiotics without other drugs or treatments, and patients with prediabetes met either impaired fasting glucose or impaired glucose tolerance or both and were free of other major medical conditions.

#### Exclusion criteria

**Fig. 1:**
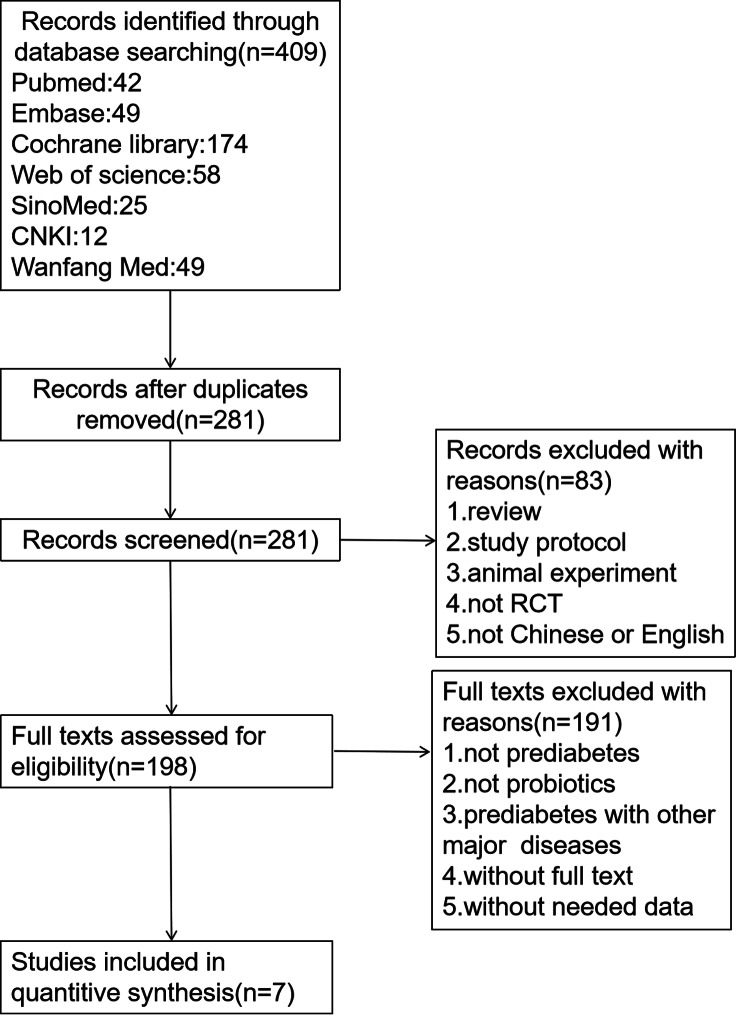
Study screening flowchart

Articles that met the following requirements were excluded: study protocols, full text not available, and not in English or Chinese. Studies that did not provide required data were also excluded. This work was performed by three researchers: two independent researchers who screened articles and a third staff member who addressed controversial issues. The study screening process is shown in Fig. 1.

### Data extraction

For the meta-analysis, the following information was summarized: (1) first author’s name, publication year and country; (2) probiotics or placebo group, number of people in each group, and age range; and (3) form of administration, type of probiotic, duration of intervention, and outcomes observed.

For the systematic review, the following related information on the included studies was summarized: (1) first author’s name and year of publication; (2) form of administration and dose in each group; (3) investigated factors and mechanisms; and (4) alterations in outcomes.

### Study quality assessment

**Fig. 2:**
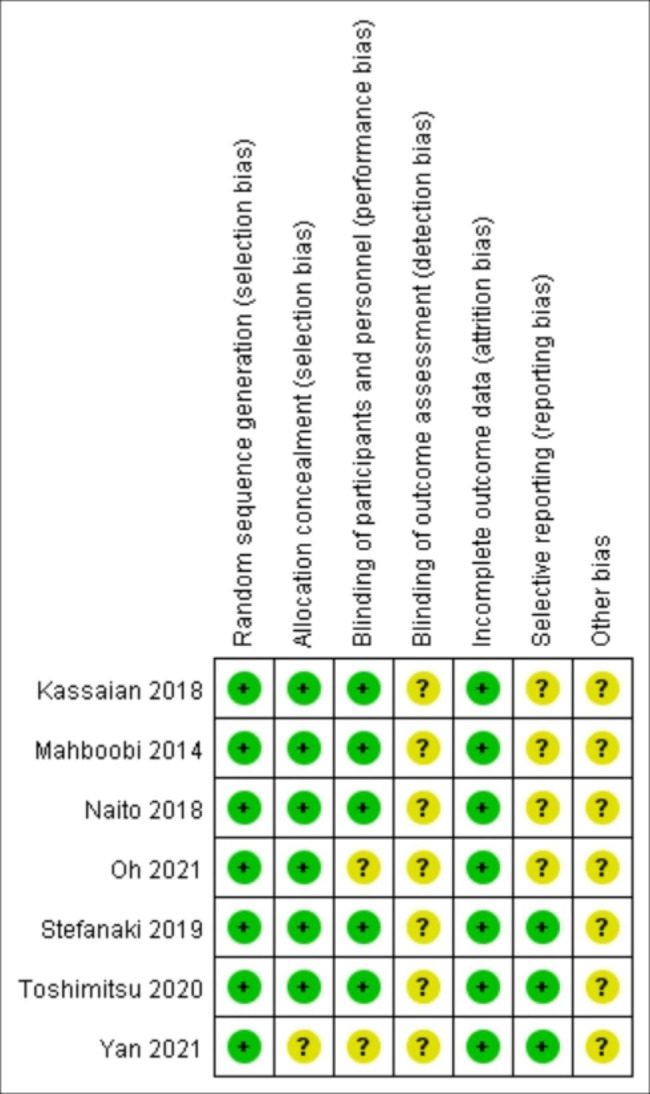
Risk of bias summary

The quality of the included studies was assessed according to the Cochrane Handbook [20]. The seven types of bias listed in the manual are selection bias, allocation concealment, implementation bias, measurement bias, follow-up bias, reporting bias, and others. The risk of bias for inclusion in the article is summarized in Fig. 2.

### Data analysis

This meta-analysis was performed using Review Manager 5.4. A fixed-effects model was used for the mean difference analysis of each study indicator. Standardized mean differences were chosen when the units and measurement methods of each indicator in the included studies were not consistent; conversely, weighted mean differences were chosen. For continuous variables, 95% confidence intervals were used. Mild, moderate, and severe heterogeneity was assessed based on I² and Chi² statistics of 0–25%, 25–50%, and 50–75%, respectively.

### Publication bias

If the number of studies included in the meta-analysis was sufficient (n ≥ 10), then the funnel plot of fasting blood glucose was plotted in Review Manager 5.4 to observe whether it was symmetrical. If the funnel plot was not symmetrical, then publication bias was indicated, and further statistical description was performed using Egger’s test. A *P* value < 0.05 suggested the existence of publication bias. Next, the indicators that caused publication bias were remedied by the trim and fill method.

## Results

### Included studies

Seven studies with a total of 460 patients were included in this meta-analysis. Of these patients, 233 were in the probiotic group, and 227 were in the placebo group. Three studies used capsules to administer probiotics, and others provided probiotics via forms of milk, yogurt, powder and sachets. Three studies treated patients with only one probiotic, whereas the rest used combinations of three or more probiotics as interventions. Details are presented in Table 1.

**Table 1:** Specific characteristics of the seven studies included in the meta-analysis

Study	Country	Sample size (experiment/control)	Age (years)	Interventions	Administration form	Probiotic strain	Duration	Outcomes
Mahboobi et al. [5]	Iran	56 (28/27)	25–65	Probiotic/Placebo	Capsule	*Lacbotacillus casei*; *Lacbotacillus acidophilus*; *Lacbotacillus phamnosus*, etc.	8 weeks	(8)
Kassaian et al. [21]	Iran	85 (27/30/28)	35–75	Probiotic/Synbiotic/Placebo	Powder	*Lactobacillus acidophilus*; *Bifidobacterium lactis*; *Bifidobacterium bifidum*, etc.	24 weeks	(1)(2)(3)(4)
Naito et al. [17]	Japan	98 (48/50)	20–64	*Lactobacillus casei* strain Shirota/Placebo	Milk	*Lactobacillus casei* strain Shirota	14–15 weeks	(1)(2)(4)(5)(6)(7)(8)
Toshimitsu et al. [22]	Japan	126 (62/64)	20–64	*Lactobacillus plantarum* OLL2712/Placebo	Yogurt	*Lactobacillus plantarum* OLL2712	12 weeks	(1)(2)(4)
Yan et al. [23]	China	72 (41/31)	35–65	Probiotic/Placebo	Capsule	*Bifidobacterium*; *Lactobacillus-acidophilus*; *Enterococcus-faecalis*	2 years	(1)(4)(5)(6)(7)(8)
Oh et al. [24]	Korea	37 (20/17)	19–70	*Lactobacillus plantarum* HAC01/Placebo	Capsule	*Lactobacillus plantarum* HAC01	8 weeks	(1)(2)(3)(4)
Stefanaki et al. [25]	Greece	17 (7/10)	12–20	Probiotic/Placebo	Sachet	*Streptococcus thermophilus*; *Bifidobacteria breve*; *Bifidobacteria longum*, etc.	4 months	(1)(2)(5)(6)

(1) = FBG; (2) = HbA1c; (3) = QUICKI; (4) = HOMA-IR; (5) = TC; (6) = TG; (7) = HDL-C; (8) = LDL-C.

### Effects of probiotics on primary outcomes

**Fig. 3:**
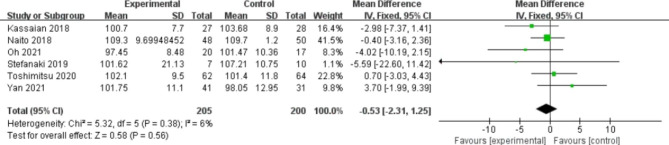
Forest plot of the effect of probiotics on FBG.

**Fig. 4:**
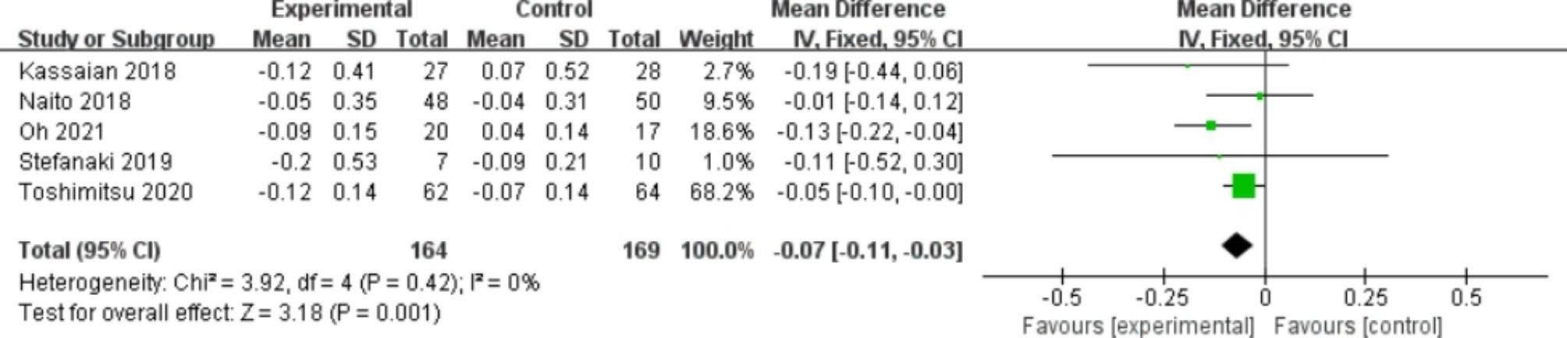
Forest plot of the effect of probiotics on HbA1c.

A total of 6 studies reported FBG (Fig. 3). No statistically significant difference was observed between the two groups (WMD, -0.53 mg/dl; 95% CI -2.31, 1.25; P = 0.56). Slight heterogeneity was found (I^2^ = 6%, P = 0.38). Regarding HbA1c, five studies mentioned it (Fig. 4). The probiotic group was prominently more effective than the placebo group (MD, -0.07; 95% CI -0.11, -0.03; P = 0.001). No heterogeneity was detected between the two groups (I^2^ = 0%, P = 0.42).

### Effect of probiotics on secondary outcomes

**Fig. 5:**
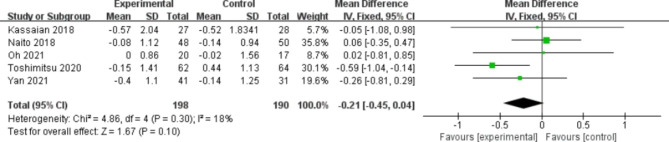
Forest plot of the effect of probiotics on HOMA-IR.

**Fig. 6:**
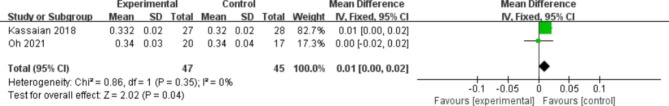
Forest plot of the effect of probiotics on QUICKI.

**Fig. 7:**
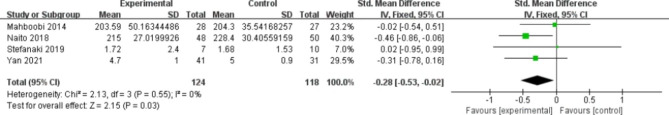
Forest plot of the effect of probiotics on TC.

**Fig. 8:**
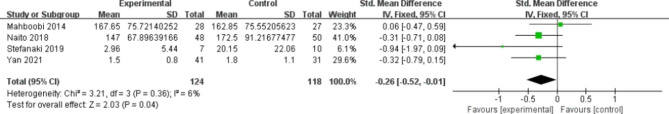
Forest plot of the effect of probiotics on TG.

**Fig. 9:**
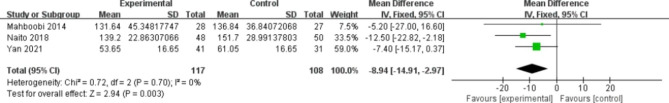
Forest plot of the effect of probiotics on LDL-C.

**Fig. 10:**
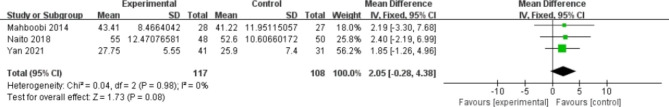
Forest plot of the effect of probiotics on HDL-C.

Five studies contained HOMA-IR (Fig. 5). No significant difference was found between the two groups, and there was a low level of heterogeneity (MD, -0.21; 95% CI -0.45, 0.04; P = 0.10; I^2^ = 18%, P = 0.30 for heterogeneity). Two studies used the QUICKI indicator (Fig. 6). The probiotic group was markedly more efficacious than the placebo group (MD, 0.01; 95% CI 0.00, 0.02; P = 0.04). No heterogeneity was observed. Four articles examined TC (Fig. 7). The probiotic group was more efficient than the placebo group (SMD, -0.28; 95% CI -0.53, -0.02; P = 0.03). No heterogeneity was observed. Four articles addressed TG (Fig. 8). A better outcome was found in the probiotic group than in the placebo group and was accompanied by subtle heterogeneity (SMD, -0.26; 95% CI -0.52, -0.01; P = 0.04; I^2^ = 6%, P = 0.36 for heterogeneity). Three studies involved LDL-C (Fig. 9). There was better efficacy in the probiotic group compared to the placebo group and a lack of heterogeneity (MD, -8.94; 95% CI -14.91, -2.97; P = 0.003; I^2^ = 0%, P = 0.70 for heterogeneity). Three studies involved HDL-C (Fig. 10). No significant differences were found between the two groups (MD, 2.05; 95% CI -0.28, 4.38; P = 0.08). There was also no heterogeneity.

### Probiotic mechanisms of action and adverse reactions

In this systematic review, we observed that probiotics could play an active role in blood glucose homeostasis in the following ways. Kassaian et al. [21] found that probiotics can promote glucagon-like peptide 1 (GLP-1) secretion from intestinal L cells to exert a hypoglycaemic effect. Natio et al. [17] discovered that probiotics could enhance pancreatic β-cell function when *Lactobacillus casei* strain Shirota-fermented milk was administered to prediabetic patients. Toshimitsu et al. [22] found that yogurt containing *Lactobacillus plantarum* OLL2712 is able to suppress chronic inflammation and alleviate insulin resistance. Yan et al. [23] administered oral probiotics to people with abnormal glucose tolerance and found that the proportion of *Lactobacillus* and *Eubacterium eligens* in the intestine of the patients was increased, indicating that probiotics could improve intestinal flora structure to a certain degree. Stefanaki et al. [25] found that probiotics not only decrease the levels of lipopolysaccharide (LPS) and proinflammatory cytokines to increase insulin sensitivity but also reduce the abundance of harmful flora related to insulin resistance and the inflammatory response. The adverse reactions in the probiotic group that occurred during the trial were all common minor gastrointestinal complications, such as flatulence, dyspepsia, dysphagia and constipation. Some articles mentioned that these minor adverse reactions were improved by continuing to take probiotics or reducing the daily doses. Specific information is presented in Table 2.

**Table 2:** Specific characteristics of the eight studies included in the systematic review

Study	Administration Dose				Factors	Mechanisms	Outcomes	Adverse Reactions	
	**Probiotics**	**Placebo**	**Probiotics**	**Placebo**	**Investigated**			**Probiotics**	**Placebo**
Mahboobi et al. [5]	Probiotic capsules	Placebo capsules	500 mg/day	500 mg/day	-	-	SBP↓	-	-
Kassaian et al. [21]	Probiotic powder	Synbiotics powder/Placebo powder	6 g/day	6 g/day	GLP-1	Promoting GLP-1 secretion from intestinal L cells;	HbA1c↓	2/27 Flatulence, dysphagia, and dyspepsia	5/28 Flatulence, dysphagia, and dyspepsia
Naito et al. [17]	Probiotic fermented milk	Placebo milk	100 ml bottle/day	100 ml bottle/day	-	Enhancing pancreatic β-cell function	1-h post-load PG↓; GA↓; HbA1c↓; TC↓; LDL-C↓; non-HDL-C↓	No serious adverse effects	No serious adverse effects
Toshimitsu et al. [22]	Probiotic yogurt	Placebo yogurt	112 g/day	112 g/day	IL-6, IL-8, MCP-1, TNF-α, hsCRP	Suppressing chronic inflammation and insulin resistance;	HbA1c↓	No serious adverse effects	No serious adverse effects
Yan et al. [23]	Probiotic capsules	Placebo capsules	2 capsules/twice daily	2 capsules/twice daily	-	Improving intestinal flora structure	Proportion of *Lactobacillus* and *Eubacterium eligens*↑	No adverse effects	3/33 Headache; Diarrhoea
Oh et al. [24]	Probiotic capsules	Placebo capsules	1 capsule/day	1 capsule/day	-	-	2 h-PPG↓; HbA1c↓	3/20	4/17
Kassaian et al. [10]	Probiotic powder	Synbiotics powder/Placebo powder	6 g/d	6 g/d	-	-	Hyperglycaemia↓; Hypertension↓; Metabolic syndrome↓ Low HDL-C↓	2/27 Mild flatulence, dysphagia, and dyspepsia	5/28 Mild flatulence, dysphagia, and dyspepsia
Stefanaki et al. [25]	Probiotic sachets	Life-style intervention	twice daily	twice daily	LPSs; FFAs; m-TORC; IL-17 A; Butyrate; GLUT-2	Decreasing LPS and proinflammatory cytokines; Regulating intestinal bacteriome; Alleviating excessive FFAs	FBG↓; HbA1c↓	Bloating, flatulence, and constipation	No adverse effects

SBP: systolic blood pressure; TC: total cholesterol; LDL-C: low-density lipoprotein cholesterol; HDL-C: high-density lipoprotein cholesterol; non-HDL-C: non-high-density lipoprotein cholesterol; GA: glycoalbumin; PPG: postprandial plasma glucose; GLP-1: glucagon-like peptide 1; IL-6: interleukin-6; IL-8: interleukin-8; MCP-1: monocyte chemotactic protein 1; TNF-α: tumour necrosis factor-α; hsCRP: hypersensitive C-reactive protein; LPS: lipopolysaccharide; m-TORC: mammalian target of rapamycin complex; IL-17 A: interleukin-17 A; GLUT-2: glucose transporter 2; FFA: free fatty acid

## Discussion

In this study, we conducted a meta-analysis on the effects of probiotics in prediabetes and concluded that probiotics showed a statistically significant improvement in HbA1c, QUICKI, TC, TG and LDL-C in prediabetes. However, there was no distinct effect on FBG, HOMA-IR, or HDL-C. These results indicated that probiotics could improve glycolipid metabolism to some extent in prediabetes. In this light, we further systematically reviewed the mechanisms of action and side effects of probiotics in prediabetes.

Probiotics are a group of active microorganisms that primarily colonize the host’s intestinal and reproductive tracts, improve the body’s microecological balance and, when supplemented in sufficient quantities, exert beneficial effects on the enteric tract. Studies have shown that gut microecosystems are distinct between healthy individuals and diseased individuals and that dysregulation of the intestinal flora is associated with metabolic diseases such as hyperglycaemia and obesity [20, 26, 27]. More specifically, in diabetic patients, the abundance of beneficial flora such as *Lactobacillus* drops, whereas the abundance of certain Gram-negative bacteria rises. Some studies have also found that in the setting of dysglycaemia, the ratio of *Firmicutes* to *Bacteroidetes* increased, as did the abundances of *Ruminococcus/Clostridium* and *Barnesiellaceae*/*E. coli/Proteobacteria* [28–30]. However, the abundance of butyric acid-producing bacteria and the ratio of *Bacteroides*/*Verrucomicrobiae* decreased substantially [31]. There is a reduction in the number of *Bacteroidetes* in the obese population [32]. In addition to symbolic differences in bacterial populations, certain specific harmful strains of bacteria are involved in the processes that lead to altered intestinal permeability, intestinal inflammation and the pathology of insulin resistance. For example, *Collinsella aerofaciens* in the intestine increases intestinal permeability and is involved in proinflammatory processes through the production of the proinflammatory cytokine interleukin-17 A; *Firmicutes* increases LPS levels in the intestine and accelerates the inflammatory response; and *Butyrivibrio crossotus* is involved in intestinal inflammation by activating rapamycin complex signalling [25]. Hence, restoring microbial homeostasis in the human gut is of great importance to health.

In the present systematic review, we found that probiotics can restore the homeostasis of the intestinal flora and regulate blood glucose homeostasis by targeting the composition of the intestinal flora, promoting the proliferation of beneficial strains and reducing the abundance of harmful strains. For instance, the populations of *Barnesiella* spp. and *Butyrivibrio crossotus* following probiotics were observably reduced, and both were implicated in hyperglycaemia and insulin resistance [25]. Nevertheless, *Lactobacillus* inducing antimicrobial production was present at a much higher proportion after intake of probiotics [23]. Jia et al. [31] found that *Clostridium butyricum* CGMCC0313.1 was able to reduce the ratio of *Firmicutes* to *Bacteroidetes* and to increase the abundance of intestinal butyric acid-producing flora and the genus *Akkermansia*. Palacios et al. [33] also found that taking capsules with a blend of multiple probiotic strains for 12 weeks increased the abundance of SCFA-producing bacteria, including *Bifidobacterium breve*, *Akkermansia muciniphila* and *Clostridium hathewayi*, and increased plasma butyric acid levels. Certain probiotics can bring about a decrease in *Firmicutes*, which is able to produce more inflammatory molecules and exacerbate the inflammatory response, improve insulin resistance and prevent the progression of type 2 diabetes [34].

Probiotics could increase the secretion of GLP-1 in the body. GLP-1 is an endogenous intestinal hormone secreted by L cells and is critical for promoting insulin secretion through the enteroglucagon effect [35]. Concretely speaking, on the one hand, GLP-1 stimulates insulin secretion from pancreatic β cells in a glucose-dependent manner and inhibits glucagon secretion by activating the GLP-1 receptor on α cells. On the other hand, it could also promote the proliferation and regeneration of β cells and inhibit their apoptosis through the G protein-coupled receptor and TCF7L2/Wnt pathway [36, 37]. Probiotics promote GLP-1 secretion through the following three pathways. First, probiotics are able to produce short-chain fatty acids (SCFAs) by fermenting dietary fibre from the diet, which can promote GLP-1 production [38]. Second, probiotics can also indirectly stimulate GLP-1 secretion through fermentation of indigestible polysaccharides [39]. Third, probiotics transform primary bile acids into secondary bile acids, which activate Takeda G protein receptor 5, after which they stimulate the secretion of GLP-1 [40]. In this systematic review, we observed that *Lactobacillus* and *Bifidobacterium* are both indirectly capable of promoting GLP-1 production [21].

Chronic low-grade inflammation is an important pathological change in the progression of diabetes [41]. Proinflammatory cytokines can induce insulin receptor substrate-1 serine phosphorylation and block the insulin signalling pathway [42]; thus, they are considered the dominant factor in the development of insulin resistance [43]. Notably, interleukin-6 secreted by T cells stimulates the production of C-reactive protein and macrophages associated with dysglycaemia [44]. Multiple articles in this systematic review have reported that probiotics can reduce inflammation levels and improve insulin sensitivity in the following ways. Probiotics directly inhibit the production of proinflammatory cytokines or indirectly reduce the abundance of the strains involved in proinflammatory processes, maintain the integrity of the intestinal epithelial cell wall and lower LPS levels to decrease inflammatory reactions. Specifically, *Lactobacillus plantarum* may increase insulin sensitivity by inhibiting the production of proinflammatory cytokines (e.g., TNF-α). We have found that administration of probiotics can markedly decrease the abundance of *Butyrivibrio crossotus* and *Collinsella aerofaciens*, both of which are engaged in the proinflammatory response; the former can activate mammalian target of rapamycin complex signalling to induce inflammation [45], and the latter is connected with the production of the proinflammatory cytokine interleukin-17 A [46]. *Bifidobacterium* spp. and *Lactobacillus casei* are able to reduce intestinal permeability and improve intestinal epithelial cell dysfunction due to glucose transporter type 2 receptor upregulation in a dysglycaemic environment [17, 40]. LPS is a constituent of the outer cell membrane of Gram-negative bacteria and stimulates the secretion of proinflammatory cytokines by binding to the TLR4/CD14 complex [47, 48]. However, probiotic supplementation was observed to markedly lower the abundance of some Gram-negative bacteria in the gut, thus reducing LPS levels [31].

Probiotics can regulate lipid metabolism to improve blood glucose homeostasis. This imbalance may result from prolonged disturbances in blood glucose metabolism leading to more low-density lipoprotein or very-low-density lipoprotein produced by excess glycogen in the liver to bring about dyslipidaemia [5]. Consequently, we considered relevant lipid indicators as secondary outcome indicators in this meta-analysis. Probiotic supplementation substantially reduced TC, TG, and LDL-C levels. By reviewing the available reports, probiotics have been shown to promote lipid metabolism generally through the following ways, among other mechanisms. One is through the enzymatic action of bile salt hydrolase of probiotics. After hydrolysis, free bile acids cannot be reabsorbed and are excreted in the faeces, thus reducing bile sterols [22]. Second, probiotics remove cholesterol by combining with it in the small intestine [49]. Third, probiotics can also incorporate cholesterol into their cell membranes to lower blood cholesterol levels [50]. Fourth, probiotics reduce cholesterol absorption by converting cholesterol into faecal sterols via cholesterol reductase, which can be excreted in the faeces [51]. Last, probiotics inhibit the resynthesis of cholesterol through their production of short-chain fatty acids [17]. The above mechanisms of the cholesterol-lowering action of probiotics have also been validated in *in vitro* experiments. In this systematic review, we noted that supplementation with *Lactobacillus casei* strain Shirota-fermented milk markedly reduced TC and LDL-C levels [17]. Apart from that, *Bifidobacterium* and *Streptococcus* bacteria have also been identified as having the ability to lower cholesterol levels [52].

In addition, several other mechanisms, such as strengthening the mucus barrier, relieving oxidative stress, increasing leptin levels and maintaining mitochondrial health have also been implicated. The mucus layer on the surface of the intestinal epithelium is composed of mucin and mucopolysaccharides, which form the first line of defence against bacterial invasion. Certain species of probiotics could reinforce the mucus barrier by increasing the expression of mucin genes and stimulating mucus secretion [53]. For instance, *Lactobacillus* spp. can stimulate MUC3 expression and MUC2 production and secretion [54, 55]. *Bifidobacterium longum* and *Lactobacillus reuteri* could increase mucus layer thickness [56, 57]. *Pediococcus acidilactici* pA1c increases the number of cupped cells, which promote the secretion of mucus glycoproteins and maintain an appropriate length of intestinal villi [58]. Oxidative stress may play a role in damaging glucose metabolism by impairing pancreatic β cells and insulin signalling pathways. Probiotics are known to alleviate oxidative stress. First, probiotics breakdown the superoxide produced by reactive oxygen species through their own antioxidant enzymes, e.g., superoxide dismutase [59]. Second, probiotics and some of their metabolites **(**glutathione, butyrate, and folate) can increase the activity of antioxidant enzymes [16, 60, 61]. Third, probiotics also act on signalling pathways (Nrf2-Keap1-ARE, NF-κB, etc.) [62–65]. Finally, probiotics can reduce the activity of enzymes related to reactive oxygen species (e.g., cytochrome P450 enzymes and NADPH oxidase) [66]. Leptin is a protein-like hormone secreted by adipose tissue. It is worth mentioning that leptin may act in both directions with insulin, which promotes the secretion of leptin; in contrast, leptin exerts a negative feedback regulation on the synthesis and secretion of insulin. Leptin can also promote the secretion of GLP-1 by activating leptin receptors. Leptin synthesized by gastric chief cells indirectly regulates the early secretion of GLP-1 through gastrin-releasing peptide [66]. Darby et al. [67] observed that supplementation with oral *Lactobacillus rhamnosus* GG induced elevated leptin levels dependent on functional Nox1 protein in the intestine. In animal experiments, it was found that *Lactobacillus* upregulated several classes of genes related to mitochondrial function in the mouse liver. In addition, *Lactobacillus* also improves the damage to mitochondrial morphological structure caused by hyperglycemia [68]. The improvement of mitochondrial health restores the β-oxidation of fatty acids, thus reducing the accumulation of fatty acids in the liver and improving glucose metabolism throughout the body [69, 70].

Probiotics also seem to be effective in children with obesity and T2DM. For obese children, probiotics may work by promoting lipid metabolism, increasing GLP-1 secretion, raising leptin levels and regulating intestinal flora homeostasis. Firstly, several studies have shown that GLP-1 agonists (e.g., liraglutide) could be effective for weight loss in pediatric patients [71, 72]. In our systematic review, probiotics were found to increase GLP-1 secretion *in vivo*, which is essential for promoting insulin secretion through the action of intestinal proinsulin. This may suggest that probiotics could treat obese children by increasing GLP-1 secretion *in vivo*. Secondly, in the development of childhood obesity, leptin acts on the hypothalamus and exerts anorexic effects to reduce weight [73]. Probiotics can also treat childhood obesity by increasing leptin levels and suppressing energy intake. Thirdly, dysbiosis of the gut microbiota is also associated with the pathophysiology of obese children [74, 75]. New evidence suggests that an increase in the ratio of *Firmicutes* to *Bacteroidetes* leads to an increase in energy extraction from the diet, triggering obesity [76]. In our systematic review, probiotics were found to reshape intestinal flora homeostasis to improve the digestion and absorption of nutrients in the intestine. Specially, *Clostridium butyricum* CGMCC0313.1 was able to reduce the ratio of *Firmicutes* to *Bacteroidetes*. This suggests that probiotics can play a beneficial role in obese children by regulating the homeostasis of the intestinal flora. In fact, several clinical studies did prove the effectiveness of probiotics treatment in obese children [77–79]. In children with T2DM, childhood T2DM begins with reduced insulin sensitivity in skeletal muscle, adipose tissue and liver [80, 81], and obesity is a major risk factor for reduced insulin sensitivity in children [82, 83]. In turn, weight loss can improve insulin sensitivity in pediatric T2DM. In a randomized controlled trial, an 8% reduction in BMI was associated with improved insulin sensitivity in obese adolescents [84]. In addition, pediatric T2DM exhibited faster islet β-cell decline and higher rates of treatment failure compared to adult T2DM [85], and supplementation with *Lactobacillus casei* strain Shirota fermented milk was found to enhance islet β-cell function in the present study.

It is also worth stating that other toxicological effects of probiotics have been found in previous studies [86, 87]. Yeast fungemia is regarded as the most serious infectious complication caused by probiotics [88, 89]. In addition, some strains of *Lactobacillus* and *Enterococcus* could convert tyrosine and histidine into biogenic amines, which may lead to nausea, vomiting, fever and other food poisoning symptoms when in excessive amounts. *Lactobacillus* could also transfer drug-resistant genes to pathogenic bacteria via splice plasmids or transposons, triggering genetic mutations and causing disease [90]. Moreover, probiotics are not intended for everyone and should be used with caution in people who are immunocompromised, in serious medical conditions, with low intestinal barrier function, or people using central venous catheters [91].

To our knowledge, this is the first meta-analysis investigating the effect of probiotics in prediabetes patients. Probiotics were found to regulate glucolipid metabolism and improve prediabetes status through multiple mechanisms of action in this study. This study provides valuable references for subsequent related studies and future clinical translation. However, there are a few important limitations that need to be acknowledged. First, the number of included studies and the number of involved cases were restricted, and the types, amounts, and dosage forms of probiotics were different among the studies, so the conclusions could be affected to some extent in this study. Second, some of the included studies lacked statistical analysis of daily diet and exercise, and the results were somewhat biased. Third, we have limited information on the quantitative-effective relationship and minimum effective dose of known beneficial probiotic strains.

Further experimental studies are needed to explore more other beneficial probiotic strains in humans and their quantitative-effect relationships to better define their role in prediabetes. Second, large-scale and strictly controlled long-term observational clinical trials should be conducted to provide more reliable data on the efficacy and safety of probiotics, and the observation period of glucolipid metabolism in prediabetic patients after discontinuation of probiotics should be extended to determine whether the efficacy of probiotics persists for a long enough period of time. Finally, some basic experiments are needed to elucidate more clearly the mechanism of probiotic action on prediabetes at the molecular level.

## Conclusion

This paper has shown that probiotics could significantly reduce HbA1c, QUICKI, TC, TG and LDL-C in patients with prediabetes. We found that probiotics have multiple mechanisms of action in regulating blood glucose homeostasis in this systematic review. Probiotics are able to adjust the flora structure, promote GLP-1 secretion, reduce inflammation levels, regulate lipid metabolism, and some other mechanisms, including enhancing the mucus barrier, alleviating oxidative stress, elevating leptin levels and maintaining mitochondrial health to delay or block the progression of prediabetes to diabetes.

## Data Availability

The original data involved in the manuscript can be obtained from references.

## List of abbreviations

FBG: fasting blood glucose
HbA1c: glycated haemoglobin A1c
HOMA-IR: homeostatic model assessment of insulin resistance
QUICKI: quantitative insulin sensitivity check index
TC: total cholesterol
TG: triglyceride
HDL-C: high-density lipoprotein cholesterol
LDL-C: low-density lipoprotein cholesterol
WMD: weighted mean difference
SMD: standardized mean difference
GLP-1: glucagon-like peptide 1
LPS: lipopolysaccharide
SCFAs: short-chain fatty acids
